# Activation of the nicotinamide N-methyltransferase (NNMT)-1-methylnicotinamide (MNA) pathway in pulmonary hypertension

**DOI:** 10.1186/s12931-016-0423-7

**Published:** 2016-08-31

**Authors:** Andrzej Fedorowicz, Łukasz Mateuszuk, Grzegorz Kopec, Tomasz Skórka, Barbara Kutryb-Zając, Agnieszka Zakrzewska, Maria Walczak, Andrzej Jakubowski, Magdalena Łomnicka, Ewa Słomińska, Stefan Chlopicki

**Affiliations:** 1 Jagiellonian Centre for Experimental Therapeutics (JCET), Jagiellonian University, Bobrzyńskiego 14, Krakow, Poland; 2 Department of Cardiac and Vascular Diseases, Jagiellonian University Medical College, John Paul II Hospital in Krakow, Pradnicka 80, Kraków, Poland; 3 Institute of Nuclear Physics Polish Academy of Sciences, PL-31342 Kraków, Poland; 4 Chair and Department of Biochemistry, Faculty of Medicine, Medical University of Gdańsk, Dębinki 1, Gdańsk, Poland; 5 Department of Toxicology, Jagiellonian University Medical College, Medyczna 9, 30-688 Kraków, Poland; 6 Chair of Pharmacology, Jagiellonian University Medical College, Grzegórzecka 16, Krakow, Poland

**Keywords:** Pulmonary hypertension, Idiopathic pulmonary hypertension, Isolated lungs, Nicotinamide N-methyltransferase, Prostacyclin, Pulmonary endothelial dysfunction, Monocrotaline

## Abstract

**Background:**

Pulmonary arterial hypertension (PAH) is associated with inflammatory response but it is unknown whether it is associated with alterations in NNMT activity and MNA plasma concentration. Here we examined changes in NNMT-MNA pathway in PAH in rats and humans.

**Methods:**

PAH in rats was induced by a single subcutaneous injection of MCT (60 mg/kg). Changes in NNMT activity in the lungs and liver (assessed as the rate of conversion of nicotinamide (NA) to MNA), changes in plasma concentration of MNA and its metabolites (analyzed by LC/MS) were analyzed in relation to PAH progression. PAH was characterized by right ventricular hypertrophy (gross morphology), cardiac dysfunction (by MRI), lung histopathology, lung ultrastructure, and ET-1 concentration in plasma. NO-dependent and PGI_2_-dependent function in isolated lungs was analyzed. In naive patients with idiopathic pulmonary hypertension (IPAH) characterized by hemodynamic and biochemical parameters MNA and its metabolites in plasma were also measured.

**Results:**

MCT-injected rats developed hypertrophy and functional impairment of the right ventricle, hypertrophy of the pulmonary arteries, endothelial ultrastructural defects and a progressive increase in ET-1 plasma concentration—findings all consistent with PAH development. In isolated lung, NO-dependent regulation of hypoxic pulmonary vasoconstriction was impaired, while PGI_2_ production (6-keto-PGF_1α_) was increased. NNMT activity increased progressively in the liver and in the lungs following MCT injection, and NNMT response was associated with an increase in MNA and 6-keto-PGF_1α_ concentration in plasma. In IPAH patients plasma concentration of MNA was elevated as compared with healthy controls.

**Conclusions:**

Progression of pulmonary hypertension is associated with the activation of the NNMT-MNA pathway in rats and humans. Given the vasoprotective activity of exogenous MNA, which was previously ascribed to PGI_2_ release, the activation of the endogenous NNMT-MNA pathway may play a compensatory role in PAH.

## Background

Nicotinamide N-methyltransferase (NNMT) transfers the methyl group from S-adenosylmethionine (SAM) to nicotinamide (NA, niacin, vitamin B3), resulting in the formation of 1-methylnicotinamide (MNA) [1]. MNA is further metabolized via aldehyde oxidase to N(1)-methyl-4-pyridone-5-carboxamide (Met4PY) and N(1)-methyl-2-pyridone-5-carboxamide (Met2PY). NNMT activity is localized mainly in the liver but has also been detected in adipose tissue, kidney, lung, skeletal muscle, heart and brain [1, 2].

Alterations in NNMT expression and activity occurs in number of diseases. Most reports are related to cancer, whereby increased NNMT activity was linked to methylation status, histone demethylation and expression of protumorigenic genes [3, 4]. Altered NNMT activity was also associated with multiple other diseases, for example, Parkinson’s disease, liver cirrhosis, metabolic syndrome and diabetes [5–8]. Interestingly, NNMT may be either a pathogenetic factor or a protective factor in these diseases. For example, NMMT in adipose tissue was proposed to contribute to insulin resistance and obesity, while NNMT in the liver was protective against diet-induced alterations in metabolism [5, 9].

A number of other reports have suggested cytoprotective effects of NNMT activity. Increased activation of the NNMT-MNA pathway has been suggested to constitute an adaptive response of skeletal muscle in COPD, protecting against oxidant stress by reducing the levels of reactive oxygen species (ROS) [10, 11]. Overexpression of NNMT improved neuronal cell survival and increased ATP concentration via mitochondrial mechanisms [12]. Finally, MNA protected against kidney tubular injury [13] and extended the life span in *C. Elegans* via an ROS-dependent signalling mechanism [14].

The mechanisms by which NNMT activity is triggered and endogenous MNA production is initiated are still not clear. We previously demonstrated that the initiation and in early hepatitis, MNA release is linked to the energy deficit/impaired redox status in hepatocytes, while in a later phase, MNA release is linked to systemic inflammation In turn, during the single bout of exercise MNA release was IL-6-dependent [15, 16].

We previously suggested that increased NNMT activity and increased endogenous MNA concentration in atherosclerosis and hepatitis correlating with increased PGI_2_ production may represent a compensatory vasoprotective response associated with vascular inflammation [16, 17].

Pulmonary hypertension is associated with a robust inflammatory response but it is unknown whether it is associated with alterations in NNMT activity and MNA plasma concentration. In the present work, we analyzed changes in the NNMT-MNA pathway in rats with induced pulmonary hypertension and in humans with idiopathic pulmonary arterial hypertension (IPAH). In rats activity of NNMT-MNA pathway was analyzed parallel to progression of pulmonary hypertension and to changes in NO- and PGI_2_-dependent function of pulmonary circulation. Progression of PAH was investigated by assessment of right ventricular hypertrophy (gross morphology), cardiac dysfunction (by MRI), lung histopathology, lung ultrastructure and ET-1 concentration in plasma, while NO-dependent and PGI_2_-dependent function was analyzed in isolated lungs. In humans idiopathic pulmonary arterial hypertension (IPAH) was diagnosed according to the guidelines of the European Society of Cardiology based on resting hemodynamics (elevation of mean pulmonary artery pressure at rest to ≥25 mmHg, increased pulmonary vascular resistance > 3 Wood units, and pulmonary wedge pressure ≤15 mmHg) when predisposing conditions such as connective tissue disease, HIV infection, congenital heart disease, portal hypertension and anorexigen exposure were excluded.

## Methods

### Animals

To induce pulmonary hypertension, male Wistar rats (180-200 g) were injected with monocrotaline (MCT, 60 mg/kg s.c., Sigma Aldrich) solution. MCT was dissolved *ex tempore* in 1 M HCl, neutralized with 1 M NaOH and diluted with distilled water. Rats at 7, 14, 21 and 28 days post-MCT injection (1 week, 2 weeks, 3 weeks, 4 weeks post-MCT groups, respectively) were used to assess endothelial function in the isolated rat lung set-up (32 rats, *n* = 8 in each group) via the following measures: concentration of endothelial mediators in plasma, right ventricular hypertrophy, right ventricular function as visualized in vivo through Magnetic Resonance Imaging, histology and ultrastructure of the lung, and survival (40 rats, *n* = 20 in both groups).

All investigations were performed in accordance with the Guide for Care and Use of Laboratory Animals published by the US National Institutes of Health, and the experimental procedure used in the study was approved by the local Animal Research Committee (53/2009).

### Characteristics of the progression of pulmonary hypertension pathology in rats injected with MCT

#### Survival

Rats were injected with MCT solution or saline (*n* = 20 for both groups), and housed in two groups with food and water *ad libitum*. The animals were weighed every day of the experiment and observed continuously.

### Assessment of right ventricular hypertrophy and collection of tissue

The rats were weighed and anesthetized with thiopental (120 mg/kg). The chest was opened and blood was taken from right ventricle into a syringe containing citrate sodium solution (3.15 %). The obtained blood samples were immediately centrifuged (12000 rpm, 10 min) and then immediately frozen and kept at -80 °C until analysis. The heart was removed from the chest wall, the right ventricle was separated from the left ventricle and septum and was weighed. Right ventricular hypertrophy was calculated as the right ventricle to body weight ratio (RVW/BW). The lungs and liver were washed in saline, immediately frozen in liquid nitrogen and kept at -80 °C until analysis.

### Assessment of histopathological and ultrastructural changes of the lungs

For histopathology, whole lungs were dissected and fixed immediately in 10 % buffered formalin (pH 7.4) for about 24 h at room temperature. They were then washed for 2 h, dehydrated through a graded ethanol series, placed in xylene (3 times for 15 min), embedded in paraffin (58 °C, 3 h), and cast into blocks. Tissue samples were cut into approximately 5 mm thick sections by microtome, placed on glass slides and stained by hematoxylin and eosin. Light microscopic examination and photographic documentation were performed using a Zeiss microscope.

For electron microscopy (EM), lung tissue was dissected and fixed immediately using a mixture of 2.5 % glutaraldehyde, 2 % freshly prepared paraformaldehyde in 0.1 mol/L cacodylate buffer at pH 7.4. The lung tissue was fixed for 4-6 h at room temperature. Subsequently, 5-10 pieces of lung tissue were gently dissected and fixed at room temperature for another 24 h. After rinsing with 0.1 mol/L cacodylate buffer (12 h), lung tissue blocks (approximately 3 × 3 × 3 mm^3^) were post-fixed, dehydrated, and embedded in Spur resin by routine procedure [18]. Lung ultrastructure was viewed and photographed using Transmission EM Jem 1200 Ex.

### Assessment of cardiac function in vivo using Magnetic Resonance Imaging (MRI)

MRI was performed under inhalation anaesthesia applied by nose cone (1-2 vol% halothane supplemented by oxygen and air). To assess right and left ventricular function in rats, we adapted a method of examination of left ventricular function in mouse described elsewhere [19, 20]. MRI system was used consisted of a 4.7 T/310 magnet (Bruker, Germany), MARAN DRX console (Resonance Instruments Ltd, UK), ID 90 mm gradients (Resonance Research, Billerica, MA, USA), and a homebuilt 8-rung quadrature RF birdcage coil. An ECG trigger unit (SA Instruments Inc., USA) was used. Halothane was used to induce and maintain anaesthesia via a nose cone (at concentrations of 3 % and 0.5-0.7 %, respectively). The rats were placed in a supine position in the probehead and their temperature was stabilized by flowing warm air at 35 °C [21].

MR imaging was performed using an ECG triggered fast gradient echo (cine-like flow compensated FLASH) sequence. For the assessment of right (RV) and left (LV) ventricular function, at least 20 frames per cardiac cycle were acquired in short axis with the following imaging parameters: echo time (TE) 2.5 ms, acquisition matrix 128x128, field of view (FOV) 40x40 mm, slice thickness 1.5 mm, number of scans (NS) 8, and flip angle set to achieve the best contrast between myocardium and blood pool (about 30°). Whole ventricles were covered slice-by-slice.

Both right and left ventricle slice volumes were evaluated using areas of endocardium delineated in Scion Image (4.0, National Institute of Health, USA). These volumes were summed to obtain end-systolic (ESV) and end-diastolic volumes (EDV) of the right and left ventricles. Ejection fraction (EF) was calculated according to the following formula: *EF* = [(*EDV* − *ESV*)/*EDV*] × 100 %.

### Assessment of the progression of endothelial dysfunction in isolated perfused rat lung

#### The isolated lung preparation

Lungs were isolated from Wistar rats weighing 200-250 g (Lod:WIST BR form Animal Laboratory of Polish Mothers Memoria Research Institute, Hospital in Łódź, Poland). In anesthetized rats (thiopental 120 mg/kg, *i.p.*), the trachea was cannulated and the lungs were ventilated with positive pressure at a rate of 80 breaths/min (VCM module from Hugo Sachs Electronic-HSE). After laparothomy, the diaphragm was cut and nadroparine at a dose of 600 I.U. was injected into the right ventricle to prevent microthrombi formation during surgery. The animals were then exsanguinated by incision of the left renal artery. The lungs were exposed via a medial sternothomy. The pulmonary artery and left atrium were cannulated via the right and left atria, respectively.

Immediately after cannulation, the lung/heart block was dissected from the thorax. Using a tracheal cannula, the isolated lung was mounted in a water-jacketed (38 °C), air-tight glass chamber (HSE), and ventilated with negative pressure. Using a peristaltic pump (ISM 834, HSE), the lungs were perfused with Krebs-Henseleit buffer (KH-buffer; composition (in mM): NaCl 118, KCl 4.7, KH_2_PO_4_ 1.2, MgSO_4_ 1.2, CaCl_2_ 2.5, NaHCO_3_ 12.5, 4 % albumin, 0.1 % glucose and 0.3 % HEPES; pH of perfusate maintained at 7.35 throughout experiment by continuous addition of 5 % CO_2_ to the inspiratory air) at a constant flow of about 12.30 ml/min. The lungs were washed with 100 ml KH-buffer and recirculated with fresh buffer in the buffer-perfused isolated lung portion of experiments. In the blood-perfused lungs, blood perfusion was initiated immediately after mounting the isolated lung in the air-tight glass chamber. The venous pressure was set between 2-5 cm H_2_O. The end-expiratory pressure in the chamber was set to -2 cm H_2_O and inspiratory pressure was adjusted between -6 and -10 cm H_2_O to yield an initial tidal volume (TV) of about 2.0 ml. Breathing frequency was set to 80 breaths/min and the ratio of inspiration length to expiration length was 1:1 for each breath. Every 5 min throughout the experiments, a deep breath with end-inspiratory pressure of -18 cm H_2_O was automatically initiated by the VCM module (HSE) to avoid *atelectasis*. The inspired air was moisturized by bubbling through water. Airflow velocity was measured using a pneumotachometer tube connected to a differential pressure transducer (HSE), from which the respiratory tidal volume was determined.

Both arterial and venous pulmonary pressures (PAP, PVP) were continuously monitored with ISOTEC pressure transducers (HSE) connected to perfusion lines on the arterial and venous sides, respectively. Lung weight was monitored using a specially designed transducer (HSE). TC, PAP, PVP and lung weight data were acquired by the PC transducer card and subsequently analyzed by Pulmodyn-pulmo software (HSE), as well as continuously recorded on Graphtec linear recorder WR 3310.

All lungs preparations were allowed to equilibrate for the first 15 min until stable baseline PAP, PVP, and TV were achieved. At this time point, lung weight (which varied considerably between experiments) was set to zero.

### Assessment of NO- and PGI_2_-dependent function in the isolated perfused rat lung

Endothelial function in the isolated perfused lungs of rats was assessed on the basis of the magnitude of NO-dependent attenuation of hypoxic pulmonary vasoconstriction (HPV), and concentration of 6-keto-PGF_1α_ in the lung effluent. In addition, 6-keto-PGF_1α_ in the plasma of MCT-injected rats was measured.

Hypoxic pulmonary vasoconstriction (HPV) was evoked via 10-min intervals of hypoxic ventilation using a mixture of 95 % N_2_ and 5 % CO_2_. Hypoxia-induced vasoconstriction, measured as changes in PAP, stabilized after 5 min to a constant level. After cessation of acute hypoxia, PAP went back to baseline levels. Ten-minute intervals of normal ventilation occurred between each HPV procedure. After the initial induction of HPV followed by normal ventilation, the procedure was repeated twice. L-NAME (300 μM) was then added to the perfusate and perfused through the lung for 10 min, followed by two additional HPV/normal ventilation cycles. The addition of L-NAME did not result in lung oedema. Although TV, PAP, PVP and weight were continuously monitored throughout the experiment, only the maximum increase in PAP (ΔPAP) elicited by HPV was used for analysis. TV, PVP and weight did not change significantly in response to acute hypoxia in the lungs of either control or MCT-treated animals.

The concentration of 6-keto-PGF_1α_ was determined in the effluent of the isolated perfused lung and, for comparison, in the plasma of rats injected with MCT. To obtain plasma, blood was taken from the right ventricle into a syringe containing citrate sodium solution (3.15 %). The obtained blood samples were immediately centrifuged (12000 rpm, 10 min) and then immediately frozen and kept at -80 °C until analysis. PGI_2_ production in the effluent from isolated rat lung and in plasma was quantified by the measurement of 6-keto-PGF_1α_ concentration using a commercially available EIA kit (R&D). Results are expressed in pg/ml.

### Measurement of ET-1 concentration in plasma

ET-1 concentration was measured using a high performance liquid chromatography tandem mass spectrometry (LC/MS) method. An HPLC Agilent 1100 (Agilent Technologies, Waldbronn, Germany) coupled to an API 2000 triple quadrupole mass spectrometer (Applied Biosystems MDS Sciex, Concord, Ontario, Canada) equipped with an electrospray ionization interface was used and measurements were performed in positive ionization mode. Chromatographic separation was carried out with an XTerra C18 analytical column (30 mm x 2.1 mm, 3.5 μm, Waters, Ireland) at 20 °C. As an internal standard, acetyl-[DTrp16]-Endothelin-1, fragment 16-21 was used.

Briefly, sample preparation consisting of the deproteinization of proteins to ethanol was used for ET-1 isolation and after centrifugation, evaporation to dryness and redissolving a sample volume of 25 μL was injected into the analytical column for peptide analysis. Chromatography was performed on a reversed phase analytical column with gradient elution using as a mobile phase acetonitrile and water with the addition of 0.01 % of formic acid. The tandem mass spectrometer was operated at unit mass resolution in the selected reaction monitoring mode, monitoring the transition of the protonated molecular ions [M + 3H]^3+^ (m/z 832) to the product ion (m/z 318) for ET-1, with m/z 887 to m/z 228 for internal standard. The peak widths of the precursor and product ions were set to 0.7 full width, half-height. Quantification analysis was performed by looking at the peak area ratio of ET-1 to IS. The results showed linearity of the calibration curves from 1 to 200 nM in Krebs-Henseleit buffer and from 21 to 220 nM in polled rat plasma. The limit of detection of the assay in SRM mode using matrix buffer was estimated at 40 pM. The limit of quantification of ET-1 in matrix buffer was established to be 1 nM. Data acquisition and processing were accomplished using Applied Biosystems Analyst version 1.4.2 software. This method is described in greater detail elsewhere [22].

### Assessment of NNMT-MNA pathway

#### Assessment of NNMT activity in the lungs and liver

To evaluate NNMT activity, tissue extracts were assayed for the enzymatic conversion of NA to MNA in the presence of cofactor S-adenosyl-L-methionine (SAM), using liquid chromatography mass spectrometry (LC/MS). Briefly, lung and liver samples were collected and washed with cold 0.9 % (w/v) sodium chloride and kept at -80 °C. The tissue was homogenized 1:9 (w/v) in cold buffer containing 150 mM KCl, 20 mM Tris, 1 mM EDTA, 1 mM dithiothreitol (pH 7.0) and 0.1 % Triton X-100 using a teflon-glass homogenizer and was centrifuged at 14000 rpm for 10 min at 4 °C. The supernatant was immediately used to assay NNMT activity. The reaction mixture consisted of 25 μl 2 mM DTT, 6.25 μl 0.8 M Tris:HCl (pH 8.6), 12.5 μl 0.8 mM NA, 25 μl 0.4 mM SAM in 0.1 mM sulfuric acid and 50 μl of enzyme preparation. The mixture was incubated for 20 min at 37 °C and the reaction was stopped with the addition of 100 μl 1.3 M HClO_4_. Blank samples were prepared using the same procedure, but the reaction was terminated immediately, without incubation. Each sample was neutralized with 3 M K_3_PO_4_, centrifuged and analyzed using LC/MS.

The LC/MS equipment included a Surveyor HPLC connected to a TSQ Vantage triple quadrupole mass spectrometer (Thermo Scientific), with heated electrospray ionization operating in positive single ion monitoring mode. The column used for separation was Synergy Hydro-RP (4 μm, 150 x 2.0 mm). The mobile phase was composed of 10 mM nanofluoropentanoic acid (NFPA) in water (buffer A) and 100 % acetonitrile (buffer B). The flow rate was 0.2 ml/min and injection volume was 2 μl. NA, MNA, and 2-chloroadenosine, used as an internal standard, were identified and quantified from ion transitions m/z 123 → 80 for NA, 137 → 94 for MNA and 302 → 171 for internal standard, as described previously [23].

#### Measurement of plasma concentration of MNA and its metabolites (Met2PY, Met4PY)

The plasma concentrations of MNA, N(1)-methyl-2-pyridone-5-carboxamide (Met2PY) and N(1)-methyl-4-pyridone-3-carboxamide (Met4PY) were determined using high performance liquid chromatography-mass spectrometry (LC/MS). An HPLC Ultimate 3000 (Dionex, Thermo Scientific, USA) coupled to a TSQ Quantum Ultra mass spectrometer (Thermo Scientific, USA) equipped with a heated electrospray ionization interface (HESI-II Probe) was used. Chromatographic separation was carried out on an Aquasil C18 analytical column (4.6 x 150 mm, 5 μm, Thermo Scientific, USA). The mobile phase consisted of acetonitrile with 0.1 % (v/v) formic acid (A) and 5 mM ammonium formate (B). The flow rate was set at 0.8 mL/min, with isocratic elution (A:B, 80/20, v/v). The mass spectrometer was operated in the positive ionization mode using selected reaction monitoring (SRM). The transitions (precursor to product) monitored were m/z 137 → 94 for MNA, m/z 153 → 110 for Met2PY and m/z 153 → 136 for Met4PY. The transitions (precursor to product) monitored for internal standards were m/z 140 → 97 for MNA-d3, m/z 156 → 113 for Met2PY-d3 and m/z 156 → 139 for Met4PY-d3. Data acquisition and processing were accomplished using Xcalibur 2.1 software. Plasma samples were prepared using deproteinization with acidified acetonitrile.

#### Immunohistochemical determination of NNMT in the lungs and liver

After anaesthesia (thiopental 120 mg/kg, i.p.), small fragments of lung and liver tissue were collected, washed in PBS solution, and then placed in 50 % OCT solution (SakuraTek, Japan) overnight for cryoprotection. Samples were then placed into OCT freezing compound and snap-frozen at -80^○^C. OCT frozen liver and lung tissues were cut into 10 μm-thick cross-sections using a Leica CM1920 automatic cryostat and placed on poly-L-lysine-covered microscopic slides (Metzel Glaser Super Frost). Collected slides were immunostained using rabbit-anti-mouse NNMT (Abcam) primary antibody, followed by Cy3-conjugated goat-anti-rabbit secondary antibody (Jackson Immuno Research). Images were acquired using an AxioCam MRc5 digital camera and an AxioObserver.D1 inverted fluorescent microscope (Zeiss), stored as tiff files and analyzed automatically using Columbus software (version 2.4.2, Perkin Elmer).

### Measurements of MNA and its metabolites in patients with pulmonary hypertension and controls

We recruited treatment naive, adult patients with newly diagnosed IPAH who were recorded in our pulmonary hypertension data base between January 2014 and April 2015. Patients who were ≥18 years old and had blood samples stored at the time of IPAH diagnosis, were eligible for the study. We excluded patients who had active infection or unstable hemodynamics defined as the need for catecholamine support. All patients were diagnosed according to the algorithm recommended by the European Society of Cardiology. To evaluate the severity of pulmonary hypertension we chose the following parameters: New York Heart Association class, 6 min walking distance, N-terminal pro-Brain Natriuretic Peptide (NT-proBNP), and hemodynamic data including cardiac index (CI) and pulmonary arterial resistance (PAR).

Patients who were referred to our centre with the suspicion of IPAH based on echocardiography and who had mPAP < 25 mmHg, PAWP ≤15 mmHg, and a cardiac index (CI) ≥ 2.5 L/min/m^2^ were taken as controls.

The venous blood samples were taken from each patient after an overnight fast in the morning on the day of diagnostic catheterization Blood samples were taken from the antecubital vein using sodium citrate (0.109 M) or EDTA as anti-coagulants and centrifuged within one hour after collection (2500 g, 20 min). Plasma samples were stored in aliquots frozen until analysis. ET-1, NT-proBNP and 6-keto-PGF_1α_ concentration in plasma were measured by ELISA (R&D), while MNA, Met2PY, Met4PY by LC/MS as described above.

The institutional ethics committee approved the registry of patients with PAH and the controls including blood sample storage and both the patients and controls signed the informed consent (approval no 66/KBL/OIL/2009). All clinical investigations have been conducted according to the principles expressed in the Declaration of Helsinki

### Statistical analysis

Results are presented as mean ± SEM or median (IQR). The normality of the results was analyzed using the D'Agostino & Pearson omnibus normality test and the Shapiro-Wilk test. To calculate statistical significance, a paired Student’s *t*-test, Mann-Whitney test or unpaired Student *t*-test was used. Post-hoc analysis was calculated using Dunn's multiple comparisons test. The survival was analyzed with the Mantel-Cox's test.

## Results

### Development of PAH induced by monocrotaline (MCT)

**Fig. 1:**
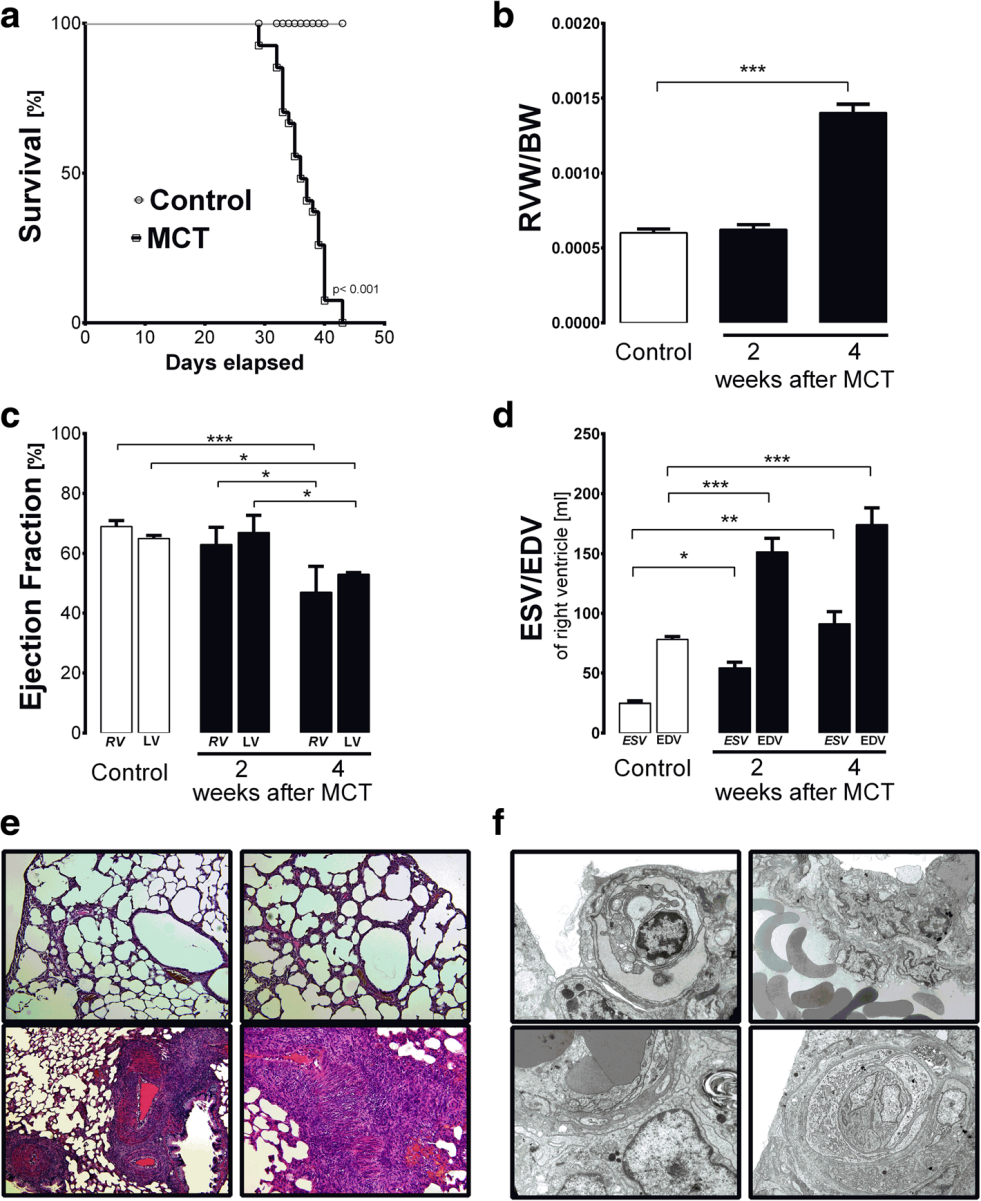
Development of pulmonary hypertension after injection of MCT in rats. **a** Survival of rats injected with MCT, with 100 % mortality at 45 days after MCT injection. **b** Significant right ventricular hypertrophy (RVW/BW) at 4 weeks after MCT injection (Control *n* = 10; MCT-treated *n* = 10 each). **c** Impairment of Ejection Fraction (EF) in the right ventricle (RV) and left ventricle (LV) at 4 weeks after MCT injection (Control *n* = 4; MCT-treated *n* = 5 each). **d** Changes in End-Systolic and End-Diastolic Volume (ESV and EDV) in rats injected with MCT showed increased ESV and EDV until 2 weeks after MCT injection (Control *n* = 4; MCT-treated *n* = 5 each). **e** Histological image of lung tissue in control rats (upper row) and 4 weeks post-MCT (bottom row). In the bottom left cross-section with two pulmonary arteries parallel to bronchioles, thickened arteries with massive hypertrophy and disorganization of vascular smooth muscle cells can be seen – one with a concentric hypertrophic artery and very small lumen; on the bottom-right picture, horizontal and oblique section of hypertrophic pulmonary artery. **f** Ultrastructure of lung from rats at 4 weeks after MCT injection. Upper-left image: degenerated endothelial cell; upper-right: activated endothelial cell with large nucleus and fibrosis of pulmonary tissue; bottom-left: pathological, multi-layered endothelial cells on thickened basal lamina; bottom-right: neovascularization in lung tissue: two endothelial cells (EC) surrounded with pericyte on thickened basal lamina. Data are presented as mean ± SEM. *P < 0.05, **P < 0.01, ***P < 0.001

As shown in Fig. 1, injection of MCT resulted in severe PAH with 100 % mortality (Fig. 1a). Four weeks after MCT, development of PAH was evidenced by right ventricular hypertrophy (RVW/BW 0.14 ± 0.01 *vs* 0.07 ± 0.01, in post-MCT and Control groups, respectively, *P* < 0.05; Fig. 1b), increased ESV (End-Systolic Volume) and EDV (End-Diastolic Volume), and a decrease in the EF (Ejection Fraction) of the right ventricle (ESV: 105.41 ± 16.07 *vs* 24.84 ± 3.27 ml, *P* < 0.05; EDV: 202.80 ± 7.34 *vs* 77.97 ± 3.91 ml, *P* < 0.05; EF: 0.48 ± 0.09 *vs* 0.68 ± 0.02 %, *P* < 0.05 for post-MCT and Control groups, respectively; Fig. 1c, d). In addition, during cardiac contraction, movement of the septum towards the left ventricle was clearly visible, supporting the presence of increased intraventricular pressure in the right ventricle compatible with the development of PAH 4 weeks after MCT injection. Left ventricle EF was only modestly affected in advanced PAH (0.53 ± 0.01 *vs* 0.65 ± 0.01 % at 4 weeks in post-MCT and Control groups, respectively, *P* < 0.05).

The progression of PAH was also evidenced by remodelling of the small pulmonary arteries, with typical thickening, muscularization and disorganization of the media of the pulmonary arterial wall associated with inflammatory cell infiltration around vessels and in the interstitial lung space (Fig. 1e). Ultrastructural signs of injury to pulmonary endothelial cells (endothelial oedema, vacuolization and degeneration), thickening of the subendothelial basal lamina (Fig. 1f), and active formation of novel pulmonary vessels were also compatible with PAH development (Fig. 1f).

### Alteration in NO- and PGI_2_-dependent function in pulmonary circulation after MCT

**Fig. 2:**
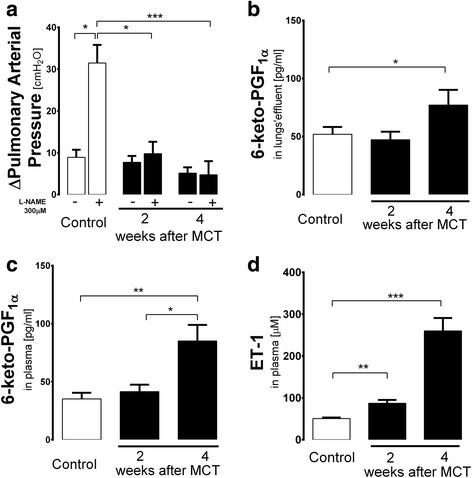
NO- and PGI_2_-dependent function in the isolated lung in PAH and systemic prostacyclin production. **a** Progressive impairment of L-NAME-induced effect on the magnitude of hypoxic pulmonary vasoconstriction (HPV) in isolated blood-perfused lungs 1-4 weeks post-MCT, with no changes in basal HPV; hypoxic ventilation with 5 % CO_2_ + 95 % N_2_ (Control *n* = 8, 2 weeks post-MCT *n* = 6, 4 weeks post-MCT *n* = 9); ΔPAP – the change in Pulmonary Arterial Pressure. **b** Lack of changes of 6-keto-PGF_1α_ concentration followed by a compensatory increase in 6-keto-PGF_1α_ in the effluents of isolated Krebs-Henseleit-perfused lungs at 2 and 4 weeks post-MCT, respectively (Control *n* = 15, 2 weeks post-MCT *n* = 12, 4 weeks post-MCT *n* = 16). **c** Changes in 6-keto-PGF_1α_ concentration: increased 6-keto-PGF_1α_ in plasma 4 weeks post MCT (Control *n* = 15, 2 and 4 weeks post-MCT *n* = 15). **d** Changes in ET-1 concentration in plasma of rats treated with MCT: increase at 2 and 4 weeks post MCT (Control *n* = 11, 2 weeks post-MCT *n* = 12, 4 weeks post-MCT *n* = 18). Data are presented as mean ± SEM. *P < 0.05, **P < 0.01, ***P < 0.001

PAH was associated with diminished NO-dependent regulation of hypoxic vasoconstriction and increased 6-keto-PGF1α release. As shown in Fig. 2, the effect of L-NAME on the magnitude of HPV in post-MCT isolated lungs was markedly diminished 2 weeks post MCT and completely abolished 4 weeks post MCT (ΔPAP after HPV + L-NAME was 4.71 ± 3.28 *vs* 31.45 ± 4.33 cm H_2_O at 4 weeks for post-MCT and Control groups, respectively, *P* < 0.01; Fig. 2a). In turn, the concentration of 6-keto-PGF_1α_ in the effluent of perfused isolated lung was unchanged at 2 weeks post MCT but was substantially increased 4 weeks post MCT (77.13 ± 13.08 *vs* 51.99 ± 6.24 pg/ml at 4 weeks for post MCT and Control groups, respectively, *P* < 0.05) (Fig. 2b). There was also an approximately 2-fold-increase in the concentration of 6-keto-PGF_1α_ in plasma 4 weeks post MCT, as compared to Control (Fig. 2c) as well as increase plasma concentration of ET-1, which is considered a reliable biomarker of PAH progression (ET-1 in plasma: 91.42 ± 7.63 and 214.95 ± 27.87 *vs* 47.95 ± 2.67 ng/ml at 2 weeks and 4 weeks for post-MCT and Control groups, respectively, *P* < 0.05) (Fig. 2d).

### Alterations in NNMT activity in the lungs and liver in MCT-induced PAH

**Fig. 3:**
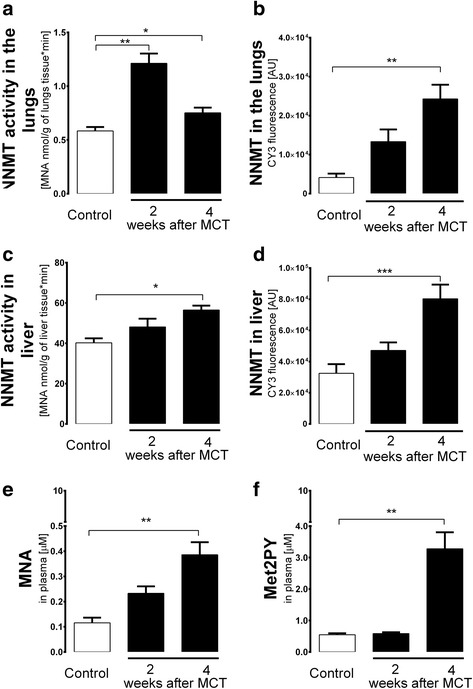
Changes in NNMT pathway in MCT-induced pulmonary hypertension. **a** Increase in NNMT activity in the lungs at 2 and 4 weeks post MCT (*n* = 4 in each group). **b** Increase in NNMT immunofluorescence in the lungs at 4 weeks post MCT (*n* = 3 in each group). **c** Increase in NNMT liver activity and **d** immunointensity at 4 weeks post-MCT (*n* = 4 in each group). **e** Increase in plasma MNA concentration at 4 weeks post MCT (Control *n* = 5, 2 weeks post-MCT n = 6, 4 weeks post-MCT *n* = 9). **f** Increase in Met2PY concentration at 4 weeks post MCT (Control *n* = 5, 2 weeks post-MCT *n* = 6, 4 weeks post-MCT *n* = 9). Data are presented as mean ± SEM. *P < 0.05, **P < 0.01, ***P < 0.001

In control rats, the activity of NNMT in the liver was almost 80-fold higher than in the lungs (40.26 ± 2.212 *vs* 0.58 ± 0.054 μmol MNA/min/mg wet tissue in liver and lung, respectively; Fig. 3a, c). NNMT activity in the liver increased progressively over the progression of MCT-induced PAH (48.10 ± 4.095 and 56.48 ± 2.183 *vs* 40.26 ± 2.212 μmol MNA/min/mg of wet liver tissue at 2 weeks and 4 weeks for post-MCT and Control groups, respectively; Fig. 3c). Similarly, immunostaining revealed progressively increased NNMT expression in post-MCT liver (Fig. 3d). In the lungs, both NNMT activity and immunostaining showed substantial increases; however, the progressive pattern and parallel increase of NNMT activity and immunostaining was less evident than in the liver (Fig. 3a, b).

### Changes in plasma concentration of MNA and its metabolites in MCT-induced PAH

MNA concentration in plasma rose over the progression of PAH and was significantly higher 4 weeks after MCT injection (0.38 ± 0.050 and 0.11 ± 0.021 μM at 4 weeks for post-MCT and Control groups, respectively, *P* < 0.05, *n* = 6-9) (Fig. 3e) while plasma concentrations of nicotinamide was stable over the course of PAH development (results not shown). Concentrations of Met2PY in plasma were significantly increased 4 weeks post MCT (Fig. 3f), while plasma concentrations of Met4PY were decreased at 2 and 4 weeks post MCT. Interestingly, the Met2PY/Met4PY ratio changed considerably along the progression of PAH. This was due to a fall in Met4PY 2 weeks post MCT, and changes in Met2PY 4 weeks post MCT (Fig. 3f). There was a correlation between MNA and PGI_2_ concentrations in plasma at 4 weeks post MCT (*r* = 0.644, *p* = 0.06, *n* = 9).

### Changes in plasma concentration of MNA and its metabolites in idiopathic pulmonary arterial hypertension (IPAH)

We enrolled 10 IPAH patients and 8 controls. Most patients with IPAH were in WHO functional class 3 (*n* = 7), the others in functional class 2 (*n* = 2) and 4 (*n* = 1). Control subjects did not have dyspnoea. The characteristics of IPAH and Control groups is presented in Table 1.

**Table 1:** Characteristics of IPAH patients and Control group

Parameter (units)	IPAH patients, n=10	Controls, n=8	*p*
age (years)	41 (37-50)	54 (37-60)	0.24
sex (M)	3 (30 %)	3 (38 %)	0.87
NT-proBNP (pg/ml)	1204 (837-2327)	63 (28.5-135,4)	0.0002
ET-1 (ng/ml)	2.54 (2.06-2.79)	0.63 (0.49-0.86)	0.0002
6MWD (m)	397.5 (345-420)	460 (420-630)	0.02
Hb (g/dl)	15.4 (14.4-16.0)	13.3 (11.8-14.4)	0.01
PAP mean (mmHg)	52 (43-56)	12 (10-14.5)	0.0004
PWP (mmHg)	6 (3-8)	7.5 (6-9.5)	0.35
RAP mean (mmHg)	5 (4-7)	2 (2-3)	0.002
CI (l/min/m^2^)	1.9 (1.4-2.3)	3.2 (2.5-3.5)	0.002
PAR (dyn*s*cm^-5^)	1111 (888-1260)	77.9 (32.1-97.6)	0.0004

*NT-proBNP* N–terminal pro-brain natriuretic peptide, *ET-1* endothelin-1, *6MWD* six minute walking distance, *Hb* hemoglobin, *PAP mean* mean pulmonary arterial pressure, *PWP* pulmonary wedge pressure, *RAP* right atrial pressure, *CI* cardiac index, *PAR* pulmonary arterial resistance

**Fig. 4:**
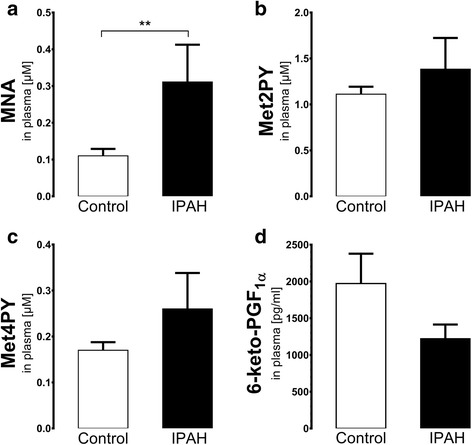
MNA and 6-keto-PGF_1α_ concentrations in plasma of patients with idiopathic pulmonary hypertension (IPAH). **a** increased concentration of MNA in IPAH. **b** lack of changes of concentrations of Met2PY between IPAH and Control groups. **c** lack of changes of concentrations of Met4PY between IPAH and Control groups. **d** lack of changes of concentrations of stable prostacyclin metabolite, 6-keto-PGF_1α_ between IPAH and Control groups. All data are presented as mean ± SEM; *n* = 8 for Control, *n* = 10 for IPAH; ** *P* < 0.01

MNA concentration in plasma was approximately 3 times higher in patients with IPAH as compared to controls (MNA: 0.31 ± 0.101 and 0.11 ± 0.019 μM, respectively, *P* < 0.01, *n* = 8-10) (Fig. 4a). Concentrations of MNA metabolites did not differ between IPAH and control groups (for Met2PY: 1.38 ± 0.339 and 1.11 ± 0.081 μM, respectively, *n* = 8-10; for Met4PY: 0.26 ± 0.078 and 0.17 ± 0.018 μM, respectively, *n* = 8-10, Fig. 4b, c). Prostacyclin production assessed as concentration of 6-keto-PGF_1α_ in plasma was also similar for IPAH and Controls (Fig. 4d).

## Discussion

In the present work, we demonstrated, to our knowledge for the first time that pulmonary hypertension is associated with the activation of the NNMT-MNA pathway in rats and in humans. We comprehensively characterized MCT-induced PAH in rats by looking at the development of right ventricular hypertrophy, right ventricular dysfunction, alterations in lung histopathology, lung ultrastructure, and ET-1 plasma concentration—all changes reported were fully compatible with the development of PAH. Taking advantage of the isolated lung set-up, we also found that PAH development was associated with impaired NO-dependent but not PGI_2_-dependent function. Most importantly, we demonstrated that over the course of PAH development, NNMT activity progressively increased in the liver and in the lungs, and that this response was associated with increased MNA concentration in plasma. In humans with newly diagnosed, treatment naive IPAH MNA plasma concentration was also significantly elevated supporting the notion that NNMT-MNA pathway is activated not only in rats but also in humans in the course of development of pulmonary hypertension.

The monocrotaline-induced model of PAH mimics key features of human PH, for example increased ET-1 concentration in plasma, and a pro-inflammatory pulmonary vascular response [24–32]. In our hands, MCT-induced PAH, was featured by progressive increase in right ventricular end-diastolic volume and a significant impairment of the right ventricular ejection fraction, as well as with gradual increase in ET-1 concentration in plasma [28, 33, 34]. Ultrastructural features of an injured pulmonary endothelium, including a thickened basal lamina, with neovascularization and fibrosis, were also compatible with PAH [35–39]. These findings are in accordance with the previously described profile of MCT-induced PAH in rats [27–29, 40] and provided a reliable background for profiling changes in NNMT-MNA pathway activity and correlating them with changes in systemic and pulmonary PGI_2_-dependent function.

Taking advantage of the isolated lung set-up we demonstrated that NO-dependent endothelial function in the pulmonary circulation was impaired while PGI_2_ release from pulmonary circulation was not changed in the early phase of PAH development and increased in advanced PAH. Previously, other authors have reported decreased or increased production of PGI_2_ during various stages of PAH development [33, 40–44]. Our results tend to show that early after MCT-injection, PGI_2_ production decreases (results not shown), while in advanced PAH, it increases [44] as a compensatory response to NO-deficient state. Interestingly, the compensatory increase in PGI_2_ production is a phenomenon often present in diseases showing impaired NO-dependent function [45]. Although we did not define the enzymatic sources of PGI_2_, in the monocrotaline-modified rat lung, it is most likely COX-2 [44, 46].

In the present work, we found that the development of PAH was associated with a progressive increase in NNMT activity/immunointensity and increased MNA concentration in plasma. The mechanism involved was not investigated but proinflammatory cytokines may be involved e.g., IL-6 that increases in PAH, and was shown to stimulate NNMT expression in the liver [25, 47]. On the other hand, it could well be that an energy-failure-related mechanism following hypoxia or metabolic stress [48] in the lung may be involved in the activation of NNMT in the lung in MCT-induced PAH [49, 50]. Increased NNMT in the lungs, most likely localized to pulmonary epithelial and endothelial cells may be also related to the increased cellular proliferation observed in the MCT model [51, 52].

As the NNMT activity in the lungs was approximately 80-fold lower than in the liver, the major source of MNA release responsible for the nearly 4-fold increase in plasma MNA concentration seen in advanced PAH may be most likely ascribed to the activation of the NMMT-MNA pathway in the liver, despite the fact that NNMT is also present in other tissues [1, 2]. Unexpectedly, only Met2PY but not Met4PY increased along with MNA, suggesting modified function of aldehyde oxidase by MCT metabolites in the liver [53, 54].

Obviously, we could not investigate the cellular source of increased plasma MNA in patients with IPAH. Nevertheless we suspect that liver is the most likely source as it is activated by pro-inflammatory stimuli present in patients with IPAH [55, 56].

We enrolled to the study only patients who were newly diagnosed. Therefore we suspect that these patients were at relatively early stage of development of pulmonary vascular disease. Most international registries show that the mean time from the onset of symptoms to diagnosis of IPAH is 18 months [57] however the time from the onset of pulmonary vascular changes to the onset of dyspnoea in humans is unknown. Given the characteristics of patients with IPAH, we could speculate that they represent relatively early stage of PAH development more or less compatible with the disease phenotype in MCT-induced PAH 2 weeks after MCT injection but not 4 weeks after MCT that represented end-stage of PAH. Accordingly, changes in MNA, Met2PY, Met4PY and 6-keto-PGF_1α_ in the comparative stage of PAH development featured by approximately two folds elevation of plasma ET-1 concentration were quite similar, displaying increased plasma MNA concentration without changes in Met2PY, Met4PY and 6-keto-PGF_1α_ plasma concentration.

Exogenous MNA possesses anti-thrombotic and anti-inflammatory activity mediated by the COX-2/PGI_2_ pathway [58, 59]. MNA had also been shown to have gastroprotective, vasoprotective, hepatoprotective and anti-diabetic properties [17, 47, 60–63]. Still, the mechanisms of PGI_2_ release by MNA remain unclear and PGI2 - independent mechanisms of MNA action has been postulated [12, 14, 64].

Although not explicitly, we have presented a relationship between NNMT-MNA pathway activity and increased plasma PGI_2_ concentration in the end-stage PAH, suggesting a putative role of the NNMT metabolite—endogenous MNA—in the stimulation of endogenous PGI_2_ production.

Indeed, in the end stage of PAH in MCT model in rats we found correlation between MNA and 6-keto-PGF_1α_ concentrations in plasma but not in early stage of MCT-induced PAH or in IPAH in patients.

## Conclusions

Progression of pulmonary hypertension in rats and humans was associated with the activation of the NNMT-MNA pathway. Given the vasoprotective activity of exogenous MNA that was previously ascribed to PGI_2_ release, the activation of the endogenous NNMT-MNA pathway described here may have a compensatory, protective role in PAH. This notion, however, still remains to be confirmed.

## Abbreviations

6MWT: 6 Minutes walking test
CI: Cardiac index
COPD: Chronic obstructive pulmonary disease
COX: Cyclooxygenase
EDV: End diastolic volume
EF: Ejection fraction
ESV: End systolic volume
ET-1: Endothelin 1
HPV: Hypoxic pulmonary vasoconstriction
IPAH: Idiopathic pulmonary arterial hypertension
MCT: Monocrotaline
Met4PY: N(1)-methyl-4-pyridone-5-carboxamide
Met2PY: N(1)-methyl-2-pyridone-5-carboxamide
MNA: 1-methylnicotinamide
MRI: Magnetic resonance imaging
NNMT: Nicotinamide N-methyltransferase
NO: Nitric oxide
NT-proBNP: N-terminal pro brain-type natriuretic peptide
PAH: Pulmonary arterial hypertension
PAP: Pulmonary arterial pressure
ΔPAP: The change in pulmonary arterial pressure
PAR: Pulmonary arterial resistance
PGI_2_: Prostacyclin
PVP: Pulmonary venous pressure
PWP: Pulmonary wedge pressure
RAP: Right atrial pressure
RVW/BW: Right ventricular to body weight ratio
